# Brain Senescence Caused by Elevated Levels of Reactive Metabolite Methylglyoxal on D-Galactose-Induced Aging Mice

**DOI:** 10.3389/fnins.2019.01004

**Published:** 2019-09-18

**Authors:** Hong Li, Ling Zheng, Chao Chen, Xiaoli Liu, Wensheng Zhang

**Affiliations:** ^1^Beijing Key Laboratory of Traditional Chinese Medicine Protection and Utilization, Faculty of Geographical Science, Beijing Normal University, Beijing, China; ^2^Engineering Research Center of Natural Medicine, Faculty of Geographical Science, Ministry of Education, Beijing Normal University, Beijing, China; ^3^The Laboratory of Vector Biology, College of Engineering, Beijing Normal University, Zhuhai, China; ^4^National and Local United Engineering Research Center for Panax Notoginseng Resources Protection and Utilization Technology, Kunming, China

**Keywords:** brain senescence, D-galactose, triosephosphate isomeras, methylglyoxal, glyoxalase I, neuroinflammation

## Abstract

Aging is a complex natural phenomenon that is manifested by degenerative changes in the structure and function of cells and tissues. D-Galactose-induced aging mice are an artificial accelerated aging model that causes memory and learning impairment, oxidative stress, and neuroinflammation. In this study, we examined the underlying mechanism of an aging mouse model induced by D-galactose. Our behavioral Morris water maze results revealed that D-galactose administration for 2 months significantly induced memory and learning impairment in C57BL/6J mice. High performance liquid chromatography (HPLC) results showed elevated levels of the metabolite methylglyoxal (MG) in D-galactose-induced aging mice. Whether and how D-galactose induces senescence by elevated levels of reactive metabolite MG remain unclear. In our study, MG mainly accumulated through the following two aspects: to increase its source, namely, the triose phosphate produced by the glycolysis pathway, and to reduce its detoxification system, namely, the glyoxalase system. Therefore, elevated MG levels may be one of the causes of brain senescence in D-galactose-induced mice. However, the molecular mechanism of the increased level of the reaction metabolite methylglyoxal requires further exploration.

## Introduction

Galactose is generally present in milk as a structural part of lactose. The main pathway of galactose metabolism in humans involves the conversion of galactose into glucose by galactokinase and galactose-1-phosphate uridyltransferase for glycolysis (Song et al., 1999). Glycolysis is a universal metabolic pathway that converts glucose into pyruvate and releases free energy to produce adenosine triphosphate (ATP). Inhibition of glycolytic enzymes, such as triosephosphate isomerase (TPI), glyceraldehyde-3-phosphate dehydrogenase (GAPDH), enolase (ENO2 and ENO3), and phosphoglycerate kinase 1 (PKM1), can induce senescence (Li et al., 2013; Lee et al., 2015). TPI catalyzes the conversion of dihydroxyacetone phosphate (DHAP) into glyceraldehyde-3-phosphate (G3P) in the process of glycolysis. During TPI-mediated catalysis, the possibility of spontaneous deamidation of this enzyme on two specific asparagine residues (15 and 71) is increased (Hipkiss, 2011). Deamination promotes enzyme dissociation and makes the enzyme a monomer that is easy to be hydrolyzed into intracellular proteins, thereby reducing the TPI enzyme activity. TPI inactivation has detrimental effects on human and animal longevity, given that increased DHAP levels are decomposed into methylglyoxal (MG) (Tajes et al., 2014).

Methylglyoxal, which is also known as pyruvic aldehyde, is mainly derived from the intermediates of triose phosphate, including G3P and DHAP, during glycolysis; furthermore, the intermediates are dephosphorylated by non-enzymatic processes or by TPI and MG synthase-catalyzed production (Marie-Julie et al., 2016; Bellahcène et al., 2017). Under normal circumstances, MG is produced by glycolysis and metabolized by the glyoxalase system into non-toxic D-lactic acid, which is minimal in the body. The glyoxalase system consists of glyoxalase I (GLO I), GLO II, and catalysis-reduced glutathione (GSH). MG and GSH produce thio-hemiacetals by non-enzymatic reactions, which catalyze the production of S-D-lactosylglutathione by GLO I. Subsequently, GLO II digests S-D-lactosylglutathione into D-lactic acid and restores GSH to achieve detoxification (Gillespie, 1979; Hovatta et al., 2005; Distler et al., 2012). GLO I, which is the rate-limiting enzyme in the reaction, plays a key role in the metabolic detoxification of MG and has been the focus of research in recent years (Thornalley, 1990; Yang et al., 2016).

However, in the pathological state of abnormal glycolysis or long-term consumption of foods with high MG content, the clearance system of the body is overloaded, causing MG to accumulate in the body (Cai et al., 2012). Metabolic disorders of MG cause severe cytotoxicity and tissue damage (Wang and Ho, 2012; Matafome et al., 2017). MG reacts with long-lived proteins to produce amino-fructose at an early stage, causing irreversible cross-linking of proteins to advanced glycation end products (AGEs) (Ramasamy et al., 2006; Genuth et al., 2015). AGEs bind to their receptors for AGEs (RAGEs), which activate intracellular inflammatory signaling pathways and produce reactive oxygen species (ROS). Nuclear factor κB (NF-κB) is phosphorylated in the nucleus and initiates the transcription of downstream genes, inducing a series of responses, including inflammation and oxidative stress (Münch et al., 2012; Hudson and Lippman, 2018).

D-Galactose-induced mouse model has been recognized worldwide and has been widely used in the study of aging and other fields. Elevated D-galactose levels cause ROS formation and decrease antioxidant enzyme activity in the brain, further inducing cognitive dysfunction, brain senescence, weakened motor function, and shortened lifespan to mimic natural aging in rodents (Li et al., 2005; Tian et al., 2005; Yoo et al., 2012; Salehpour et al., 2017). Therefore, whether and how D-galactose induces senescence by elevated levels of reactive metabolite MG remain unclear.

In the current study, we investigated the underlying mechanism of D-galactose-induced brain senescence. Results showed that long-term administration of D-galactose induced memory and study impairment. Furthermore, MG accumulation due to reduced TPI activity and impaired MG detoxification resulted in the gradual development of D-galactose-induced aging mice into the core characteristics of aging. Given that elevated MG can generalize the main features of senescence, the possibility that elevated MG levels may be a cause of senescence on D-galactose-induced aging mice is increased.

## Experimental Procedures

### Reagents

D-Galactose (purity, >99.5%), MG (40%), and perchloric acid (PCA) were obtained from Sigma-Aldrich (St. Louis, MO, United States). 5-Methylquinoxaline (5-MQ) and 2-methylquinoxaline (2-MQ) were purchased from Thermo Fisher Scientific (Waltham, MA, United States). *o*-Phenylenediamine (*o*-PD) was purchased from Avantor Performance Materials (Shanghai, China). Acetonitrile (HPLC grade) was acquired from Fisher (Thermo Fisher Scientific, Waltham, MA, United States). The primary antibodies directed against fructose-6-phosphate kinase (PFK), TPI, GAPDH, pyruvate kinase 1/2 (PKM1/2), PKM2, lactic dehydrogenase (LDHA), NF-κB, and β-actin were obtained from Cell Signaling Technology (Danvers, MA, United States). The remaining primary antibodies used in this study were as follows: GLO I (Santa Cruz Biotechnology, Santa Cruz, CA, United States) and RAGE, interleukin-1β (IL-1β), and tumor necrosis factor-α (TNF-α) (Abcam, Cambridge, MA, United States). The secondary antibodies, namely, goat anti-mouse and goat anti-rabbit, were purchased from GE Healthcare (Buckinghamshire, United Kingdom).

### Animals and Treatment

Three-month-old male C57BL/6J mice (SPF grade, male, 45 ± 5.0 g) were obtained from Beijing WTLH Biotechnology Co., Ltd. The animals were acclimated to the laboratory environment for 2 weeks before the experiment. The animals were housed under standard specific pathogen-free conditions (24 ± 2°C, 45–55% humidity, and 12 h light/dark cycle) with free access to food and water. All animal procedures were conducted in accordance with the Institutional Animal Use and Care Committee of Beijing Normal University and adhered to the “Guide for the care and use of laboratory animals” (Clark et al., 1997).

The mice were divided randomly into two groups, namely, control and model groups, with 15 mice each. The mice in the D-galactose model group were administered with a daily subcutaneous injection of D-galactose (100 mg/kg/day) at the neck for 10 consecutive weeks. Meanwhile, the normal control group was administered with an equivalent volume of saline by using the same method.

### Morris Water Maze (MWM) Test

After D-galactose treatment for 2 months, the spatial learning, and memory of the aging mouse model induced by D-galactose were analyzed through the MWM test (D’Hooge and De Deyn, 2001; Vorhees and Williams, 2006). Briefly, in the hidden platform test performed at days 1 to 7, a platform was placed at the center of a supposed quadrant. The mice were subjected to two trials per day for 5 consecutive days. During each trial, the mice were placed in the maze at four different assigned points and allowed to swim for 90 s. The escape latency was recorded by a software upon mounting the platform. When a mouse failed to reach the platform within 90 s, it was guided to the platform, and the escape latency was recorded as 90 s. In both situations, the mice were allowed to rest on the platform for 15 s and subsequently placed in the home cage. The platform was then removed in the spatial probe test at day 6. The mice were released from the opposite quadrant and allowed to swim freely for 60 s. All experiments were conducted at approximately the same time daily.

### Western Blot

Approximately 20 mg of cerebral cortex tissues was carefully dissected and lysed in 10 volumes (wt/vol) of RIPA buffer containing protease inhibitors or a protein phosphatase inhibitor (Roche, Basel, Switzerland). After centrifugation at 13,000 × *g* for 10 min at 4°C, the supernatant was preserved. Protein concentrations were determined by BCA assay. The protein concentration of each sample was the same as the lysate buffer. The samples were loaded into the SDS-PAGE and transferred onto nitrocellulose membranes (295 mA, 1.5 h). After incubation with 5% skim milk powder at room temperature for 1.5 h, the primary antibodies against PKM1/2 (1:2000), PKM2 (1:2000), PFK (1:2000), GLO1 (1:2000), β-actin (1:2000), TPI (1:2000), LDHA (1:2000), GAPDH (1:2000), RAGE (1:1000), NF-κB P65 (1: 500), and IL-1β (1:1000) were used. β-actin was used as the loading control for general protein contents. The source-matched secondary antibodies were used, and the membranes were scanned by Odyssey 9120 (LI-COR, Inc.). Then, bands were analyzed by the Odyssey software (LI-COR, Inc.).

### TPI and GAPDH Activity Assays

Triosephosphate isomerase and glyceraldehyde-3-phosphate dehydrogenase activities in the cerebral cortex were monitored using commercially available kits (Abcam, Cambridge, MA, United States) in accordance with the manufacturer’s instructions. All data were normalized against the total protein content.

### GLO I Activity Assays

Glyoxalase I enzymatic activity in the cerebral cortex was assessed following a published procedure with some modifications (McLellan and Thornalley, 1992; Morcos et al., 2008). In brief, approximately 20 mg of frozen cerebral cortex tissues was homogenized with 10 volumes (wt/vol) of saline solution. After centrifugation at 12,000 × *g* for 10 min at 4°C, the supernatant was analyzed for protein content (BCA assay), and its enzymatic activity was determined according to spectrophotometric analysis. The standard assay mixture containing 2 mM of MG, 1 mM of GSH, and 100 mM of KH2PO4 in pH 6.6 was equilibrated. Before initiating the reaction by adding the supernatant (10 μg of protein) to the assay mixture, the mixture was allowed to stand for at least 5 min to ensure the equilibration of hemithioacetal formation. The reaction was initiated by adding the supernatant to monitor the increase in absorbance at 240 nm for 5 min at 25°C, indicating the formation of S-D-lactoylglutathione. One unit of activity is defined as the formation of 1 μmol of S-D-lactoylglutathione/min/mg protein.

### Determination of MG by HPLC

Methylglyoxal was determined by HPLC in accordance with a published procedure with modifications (Zhu et al., 2014). In brief, approximately 20 mg of cerebral cortex tissues was homogenized with 10 volumes (wt/vol) of cold PBS and sonicated (20 × 5 s) on ice. The supernatant was collected by centrifugation at 13,000 × *g* for 10 min at 4°C. The supernatant was incubated with 0.5 mol/L of PCA, 1 mmol/L of *o*-PD, and 25 μmol/L of 5-MQ for 24 h at 37°C in the dark to form the specific quinoxaline derivate, 2-MQ. The sample was further centrifuged at 13,000 × *g* for 10 min at 4°C. Subsequently, the supernatant solutions were filtered with a 0.22 μm membrane. MG was quantified by the 2695 HPLC system (Waters Corp., Milford, MA, United States) with a Diamonsil-C18 column (5 μm, 250 mm × 4.6 mm, Dikma, CA, United States). The samples were allowed to run with 40% acetonitrile. Each sample (20 μL) was run for 15 min at a flow rate of 1.0 mL/min. For other analyses, ultraviolet wavelength detection was set at 315 nm, and 5-MQ was used as an internal reference.

### Measurement of Lactic Acid, Pyruvate, and Oxidative Status Parameters

Lactic acid, pyruvate, SOD, GSH-Px, and MDA levels in the cerebral cortex were monitored using commercially available kits (Nanjing Jiancheng Bioengineering Institute, China) in accordance with the manufacturer’s instructions. All data were normalized against the total protein content.

### Statistical Analysis

All results were presented as mean ± standard error of the mean and analyzed by the SPSS software (version 20.0, IBM, Chicago). The significance of comparisons between the two groups was determined by independent sample *t*-test. Furthermore, statistical significance was considered at *p* < 0.05.

## Results

### Daily Behavior and Cognitive Impairments Induced by D-Galactose Administration

**FIGURE 1:**
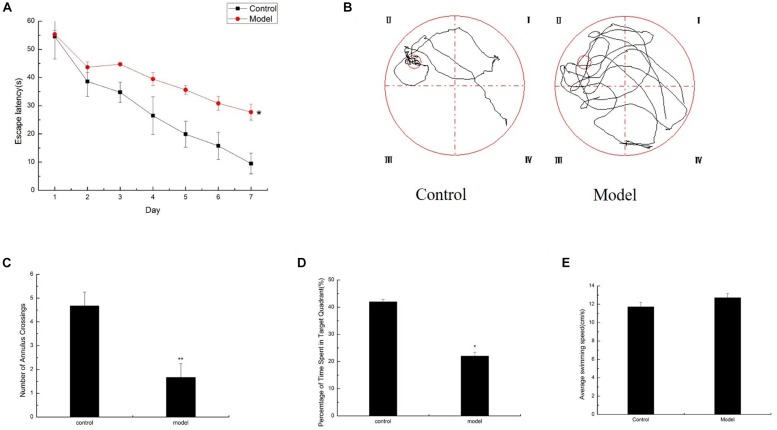
Cognitive impairments induced by D-galactose administration. Spatial learning/memory was tested with the MWM in mice at 6 months of age (*n* = 10/group). **(A)** Escape latency of the hidden-platform test (day 1–7). **(B–D)** Mean annulus crossings and time spent in the target quadrant and the swimming trace in the probe test. The small circle on the lower right panel represents the former location of the platform. **(E)** Average swimming speed. ^∗^*p* < 0.05, ^∗∗^*p* < 0.01.

Significant difference in daily behavior was observed between the control and model mice. The model group possessed an obvious aging appearance. The action response of the model mice was significantly slow, and the body was slightly bent and easily depilated. The control mice appeared noticeably better than the model mice. Meanwhile, the spatial learning and memory capacity in the aging mouse model induced by D-galactose were assessed by the MWM test. The mean escape latency of the model mice was markedly longer than that of the control mice in the hidden platform test (Figure 1A). After the space exploration test, we used the spatial probe test to detect the memory capacity and spatial exploration ability of the mice. The model mice randomly swam in the tank without knowing the target location, whereas the control mice preferentially searched for the target quadrant (Figure 1B). The number of annulus crossings and the time spent in the target quadrant was notably higher in the control mice than in the model mice (Figures 1C,D). Swimming speeds of the mice between the two groups exhibited no difference (Figure 1E), and implying that the cognitive dysfunction of model mice was not due to motor and visual impairments. Compared with the control mice of the same age, the model mice exhibited severe cognitive impairments.

### Changes in the Expression of Glycolysis by D-Galactose Administration

**FIGURE 2:**
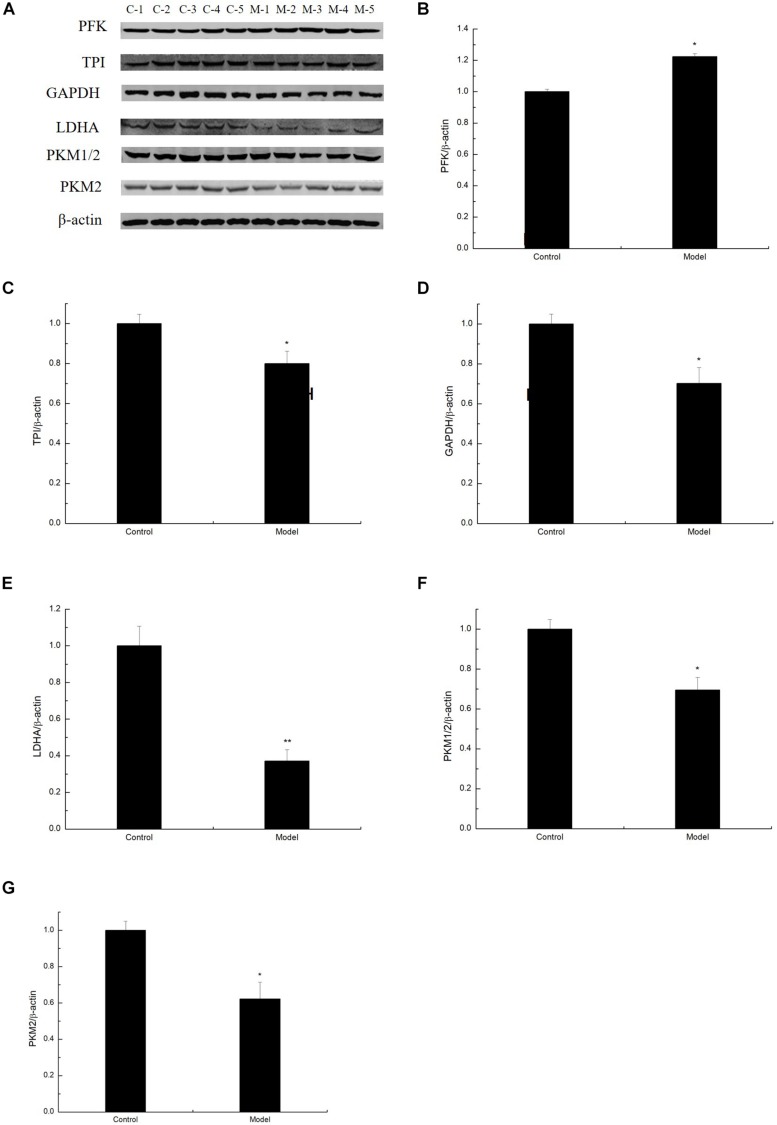
Changes in the expression of glycolysis by D-galactose administration. **(A)** Representative Western blot results of PFK, TPI, GAPDH, LDHA, PKM1/2, and PKM2 expression levels in the cortex extracts. **(B–G)** Quantification of PFK, TPI, GAPDH, LDHA, PKM1/2, and PKM2. The data are presented as means ± SD (*n* = 10), and all experiments were performed in triplicate. ^∗∗^*p* < 0.01, ^∗^*p* < 0.05 compared with the control group.

**FIGURE 3:**
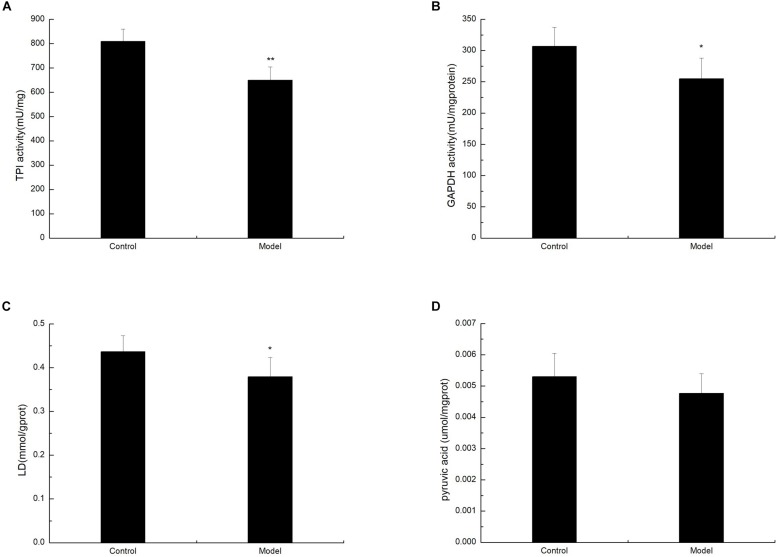
Changes in TPI, GAPDH, lactic acid, and pyruvate levels after D-galactose administration. The data are presented as means ± SD (*n* = 10), and all experiments were performed in triplicate. ^∗∗^*p* < 0.01, ^∗^*p* < 0.05 compared with the control group. **(A)** The TPI activity was decreased after administration of D-galactose in the cortex extracts. **(B)** The administration of D-galactose in the cortex extract resulted in a reduced change in GAPDH activity. **(C)** D-galactose administration results in a decrease in lactic acid content. **(D)** There no changes in pyruvate content after D-galactose administration.

To determine whether D-galactose affects glycolysis expression in the aging mouse model, we examined the expression of key enzymes during glycolysis in the mouse cortex by Western blot. The experiment showed that the expression of PFK in the model group was notably elevated compared with that of the control group (Figures 2A,B). However, the expression levels of TPI, GAPDH, LDHA, PKM1/2, and PKM2 in the model group were significantly lower than those in the control group (Figures 2A,C–G). Meanwhile, TPI and GAPDH activities were observably decreased (Figures 3A,B). The expression levels of lactic acid in the model group markedly decreased compared with those in the control group (Figure 3C), but no significant difference was observed in pyruvate expression levels between the control and model groups (Figure 3D). These data suggested that D-galactose treatment caused the activation of PFK and inactivation of TPI in the mouse cerebral cortex, resulting in an unbalanced DHAP/G3P flux, which may contribute to the conversion of a large amount of triose phosphate into MG. Thus, GAPDH, PKM, and LDHA expression levels were decreased, further causing decreased lactic acid concentration. The constant expression of pyruvate may cause normal oxidative phosphorylation, resulting in increased oxygen stress and further acceleration of aging.

### D-Galactose Administration Elevated MG Levels but Decreased MG Detoxification

**FIGURE 4:**
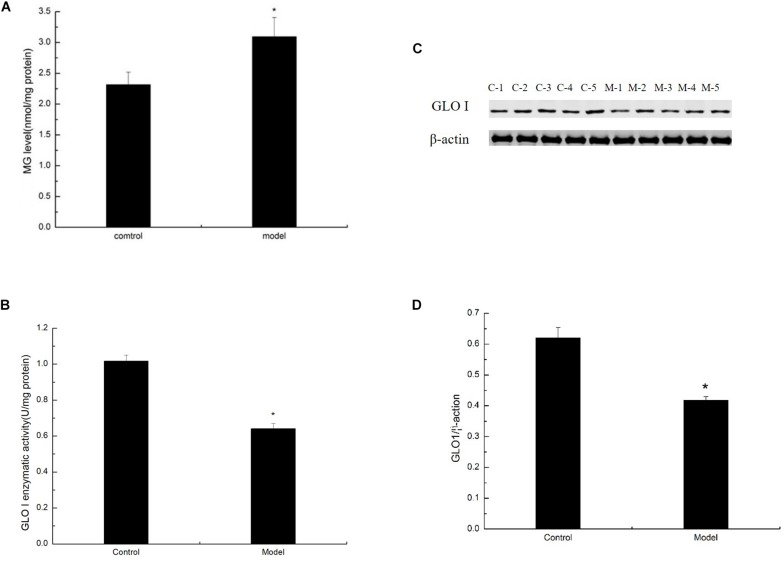
D-Galactose administration elevated MG levels but decreased MG detoxification. **(A)** MG levels were quantified by HPLC and normalized to total protein content (nmol/mg protein; *n* = 6). **(B)** Representative Western blot of GLO I expression in the cortex extracts. **(C)** Quantification of GLO I. **(D)** Enzyme activity of GLO I expression in the cortex extracts. **(B,C)** Quantification of RAGE. The data are presented as means ± SD (*n* = 6), and all experiments were conducted in triplicate. ^∗^*p* < 0.05 compared with control group.

Methylglyoxal can cause various diabetic complications and neurological lesions. In the study of nerve damage, MG cytotoxicity has become the focus of research (Fukunaga et al., 2004; Kim et al., 2012; Yan et al., 2012). To detect the conversion of large amounts of trisaccharide phosphate into MG, we estimated the level of MG in the D-galactose-induced aging mouse model by HPLC assay. The level of MG was prominently increased in the cortex of the model mice (Figure 4A). MG in the living body is mainly degraded by the glyoxalase system, in which GLO I is the rate-limiting enzyme. To explore the effect of GLO I on MG detoxification, we analyzed the expression and activity of GLO I in the cortex. As shown in Western blot, GLO I expression in the model mice markedly decreased compared with that in the control mice (Figures 4B,C). Consistently, the GLO I activity in the model mice was markedly attenuated compared with that in the control mice (Figure 4D). These findings suggested that MG detoxification on the D-galactose-induced aging mouse model was significantly reduced, resulting in an increased MG accumulation in the cortex of senescent mice induced by D-galactose.

### D-Galactose-Induced Aging Mice Exhibited an Elevated Inflammatory Response

**FIGURE 5:**
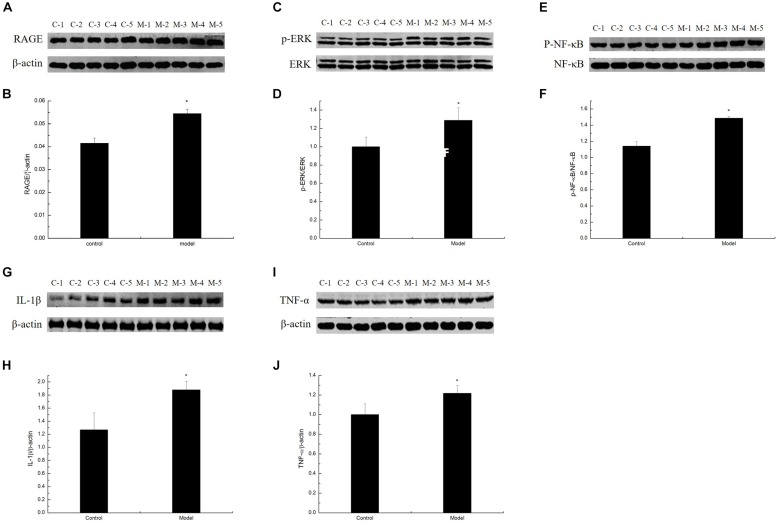
D-Galactose-induced aging mice exhibited elevated inflammatory response. **(A,C,E,G,I)** Representative Western blot of RAGE, ERK, NF-κB, IL-1β, and TNF-α expression levels in the cortex extracts. **(B,D,F,H,J)** Quantification of RAGE, ERK, NF-κB, IL-1β, and TNF-α. The data are presented as means ± SD (*n* = 10), and all experiments were performed in triplicate. ^∗^*p* < 0.05 compared with the control group.

To study whether MG affects the AGE–RAGE pathway, we further determined RAGE expression in the cortex of the D-galactose-induced aging mouse model by Western blot. Results showed that RAGE expression in the model was markedly increased compared with that in the control mice (Figures 5A,B). By binding to its receptor RAGE, AGEs can activate oxidative stress and eventually activate the redox-sensitive transcription factor NF-κB, resulting in increased levels of TNF-α, IL-1β, and other inflammatory cytokines and an impaired neurological function in the brain of aging individuals. To confirm the inflammatory response induced by AGE–RAGE interaction in the cortex of the D-galactose-induced aging mouse model, we examined the ERK, NF-κB, TNF-α, and IL-1β expression in the mouse cortex by Western blot. ERK, NF-κB, TNF-α, and IL-1β expression levels in the model mice were elevated compared with those in the control mice (Figures 5C–J; Ali et al., 2015). These data suggested that the inflammatory response in the aging mouse model induced by D-galactose was elevated compared with that in the normal mice.

### D-Galactose-Induced Aging Mice Induced Oxidative Damage

**FIGURE 6:**
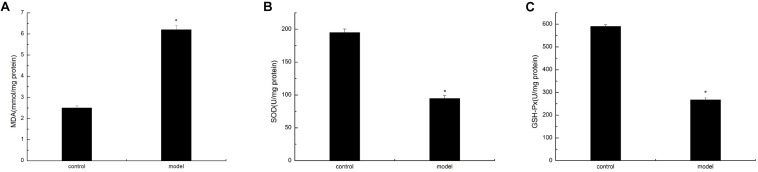
D-Galactose-induced aging mice caused oxidative damage. The data are presented as means ± SD (*n* = 10), and all experiments were performed in triplicate. ^∗^*p* < 0.01 compared with the control group. **(A)** The MDA level was increased after administration of D-galactose in the cortex extracts. **(B)** Reduction of SOD activity in mouse cortex after D-galactose injection. **(C)** D-galactose administration results in a decrease in GSH-PX content.

To verify the oxidative damage induced by AGE–RAGE interaction in the cortex of the D-galactose-induced aging mouse model, we analyzed the MDA content and SOD and GSH-Px activities. The experimental results showed that the D-galactose-induced aging mouse model possessed significantly low SOD and GSH-Px activities compared with the control group. By contrast, the MDA content in the cortex was significantly enhanced in the model group than in the normal group (Figure 6). These findings suggested that oxidative damage in the D-galactose-induced aging mouse model was significantly enhanced compared with that in normal mice.

## Discussion

Herein, we found that elevated MG levels may induce the progressive development of the main characteristics of senescence in D-galactose-induced aging mice. An aging mouse model induced by D-galactose exhibits severe cognitive impairments, neuroinflammation, and oxidative stress (Lu et al., 2006; Wu et al., 2008; Zhang et al., 2013; Rehman et al., 2017). Consistent with previous studies, after 3 months of D-galactose injection, mice showed the same appearance characteristics as those of aging mice, such as dull appearance, a slight bow, and sluggish behavior. Furthermore, D-galactose-induced aging mice demonstrated severe memory and learning impairments on the basis of the MWM test (Figure 1).

The current paradigm for D-galactose-induced aging mice is that oxygen stress and neuroinflammation are the underlying causes of brain senescence, leading to elevated MG levels, possibly due to unbalanced DHAP/G3P flux through glycolysis. During glycolysis, TPI can catalyze the conversion of triose phosphate isomers between DHAP and G3P. Compromised triose phosphate metabolism is induced by insufficient TPI activity in the tissues of aged rats (Braidy et al., 2011). Meanwhile, GAPDH, an enzyme that converts the NAD^+^-dependent oxidation of G3P into 1,3-diphosphoglycerate, undergoes MG-induced glycosylation, and inactivation (Hipkiss, 2011). In the current study, mice treated with D-galactose exhibited increased PFK levels in the body, which further increased DHAP and G3P (Figures 2A,B). In addition, the TPI expression in D-galactose-induced aging mice decreased, resulting in the spontaneous deamidation of TPI and the inability of DHAP to convert into G3P, which induced unbalanced DHAP/G3P flux and accelerated MG accumulation (Figures 2A,C). Given that a large amount of triose phosphate was used to generate MG, a small amount was used for subsequent glycolysis, and the expression of GAPDH, PKM, and LDHA was limited, further causing decreased glycolysis and lactic acid levels (Figures 2, 3). As the amount of pyruvate remained unchanged, pyruvate caused the acceleration of oxidative phosphorylation, leading to increased oxidative stress, and further aging (Figure 3D).

We also found that elevated MG in an organism is due to impaired detoxification on D-galactose-induced aging mice. The glyoxalase system, which widely exists *in vivo*, can effectively metabolize MG into non-toxic D-lactic acid, thereby maintaining MG at a low level. The primary detoxification step is the catalytic action of GLO I in the glyoxalase system (Rabbani et al., 2016). The role of GLO I in aging and life span is extensively reported. In *Caenorhabditis elegans* (*C. elegans*) mitochondria, GLO I activity significantly decreases with age and causes the accumulation of MG and oxidative stress, further inhibiting the expression and activity of GLO I. Overexpression of the glyoxalese-1 homolog (CeGly) in *C. elegans* reduces MG damage to mitochondrial proteins and prolongs *C. elegans* lifespan, whereas knocking out CeGly demonstrates the opposite reaction (Morcos et al., 2008; Xue et al., 2011). In endothelial cells, GLO I overexpression completely prevents high-glucose-induced MG accumulation and AGE formation (Shinohara et al., 1998). Through our testing, we concluded that MG accumulation is mainly due to both sources and detoxification (Figure 4). Furthermore, the expression of the GLO I gene in the brain of SAMP8 aging mice was significantly downregulated compared with that in the normal mouse SAMR1 by mRNA sequencing (Zhang et al., 2017). Hence, understanding the molecular mechanisms regulating the production and detoxification of MG may provide an important way to delay aging in the future.

Methylglyoxal is an efficiently active dicarbonyl compound and is ubiquitous in the human body (Allaman et al., 2015). Endogenous MG can be generated through various pathways in the body, such as oxidation of carbohydrates in the polyol pathway, lipid peroxidation of cell membranes, and oxidation of amino acids (Kalapos, 2008, 2013). Most endogenous MGs in cells are mainly derived from glucose and fructose metabolism (Wang and Chang, 2010; Liu et al., 2011). MG easily reacts with proteins, lipids, and nucleic acids by non-enzymatic reactions to produce AGEs. The combination of AGEs and their receptor RAGE can activate NADPH oxidase to increase the production of ROS in cells and promote the expression of the inflammatory nuclear transcription factor NF-κB, further leading to oxygen stress and inflammation (Li et al., 2005; Coker and Wagenknecht, 2011; Ali et al., 2015). Under normal physiological conditions, the concentration of MG in human cells is approximately 2–4 μM (Phillips and Thornalley, 1993). When the concentrations of its precursors increase, hyperglycemia, impaired utilization of glucose, TPI deficiency, and high MG levels can occur (Ahmed et al., 2003). Excess carbohydrates, such as glucose or fructose, in patients with Parkinson’s disease and type 2 diabetes result in increased MG production, leading to mitochondrial dysfunction (Hipkiss, 2014; Moraru et al., 2018). In the current research, we determined that the long-term administration of D-galactose, which serves as a monosaccharide, can also generate MG accumulation in mice (Figure 5A).

**FIGURE 7:**
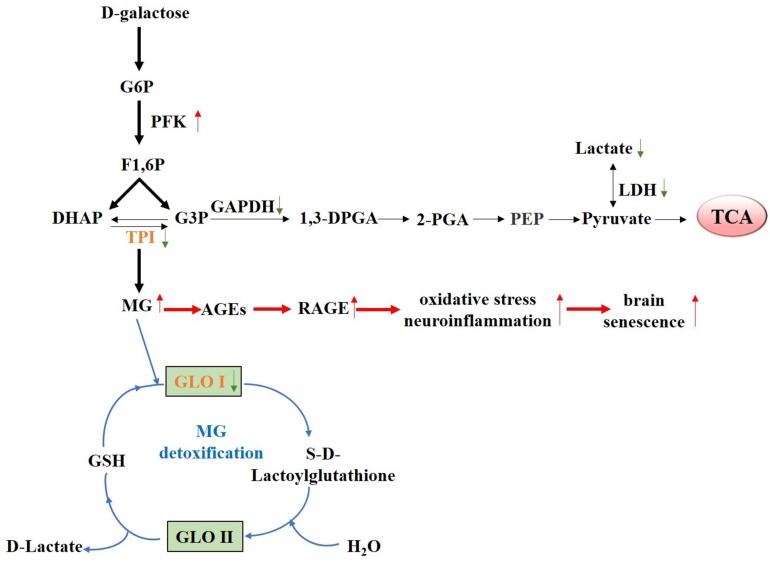
Proposed schematic representation of D-galactose-induced senescence. Schematic diagram of the changes of various D-galactose-induced enzymes, especially TPI and GLO I, causing elevated MG level. Elevated MG level further activated the AGE–RAGE signaling pathways, resulting in oxidative stress, and neuroinflammation.

**FIGURE 8:**
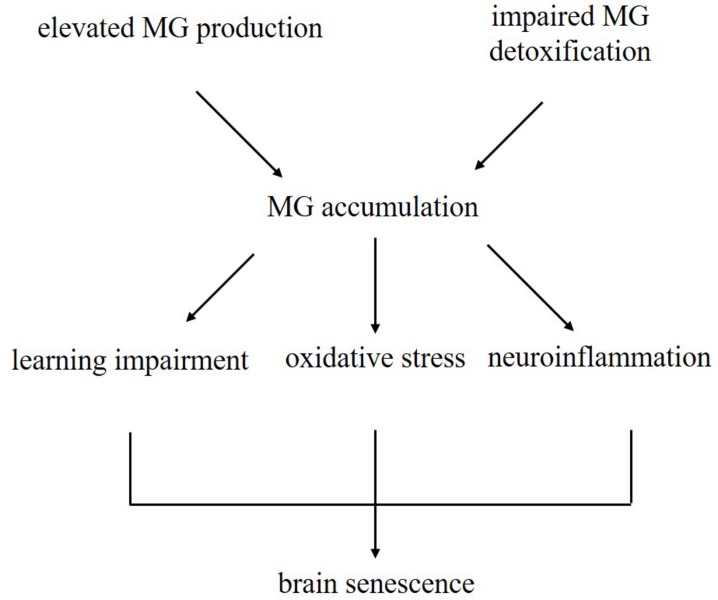
Schematic illustrating the current dominating paradigm that elevated MG levels due to increased production and impaired detoxification may be one of the causes of brain aging in D-galactose-induced aging mice.

In summary, our results demonstrated that chronic D-galactose administration induces changes in various enzymes, especially TPI and GLO I (Figure 7). Furthermore, MG concentration increased, thereby activating the AGE–RAGE signaling pathways and resulting in oxidative stress and neuroinflammation (Figure 8). The data presented here indicated that the physiological levels of TPI and GLO I play an important role in regulating organismal physiology, providing potential targets and new ideas for delaying aging, and related diseases. Why spontaneous decarboxylation of TPI and reduced activity of GLO I occur in aging individuals remains to be further explored.

## Data Availability

All datasets generated for this study are included in the manuscript and/or the supplementary files.

## Ethics Statement

The animal study was reviewed and approved by the Institutional Animal Use and Care Committee of Beijing Normal University.

## Author Contributions

HL conceived the experiments, performed the majority of the experiments, analyzed the majority of the data, and wrote the manuscript. LZ performed the feeding and management of mice. CC performed the bioinformatics analyses. XL performed the MWM test and analyzed the data. WZ provided the valuable resources, helped to design some of the experiments, and revised the manuscript. All the authors reviewed the manuscript and approved its contents and submission.

## Conflict of Interest Statement

The authors declare that the research was conducted in the absence of any commercial or financial relationships that could be construed as a potential conflict of interest.
